# Supplementary material for: RNA-Seq Analysis of Testes from Mice Exposed to Neodymium Oxide
Source: Toxics. 2023 Nov 22;11(12):952. doi: 10.3390/toxics11120952 (PMC10748220; doi:10.3390/toxics11120952)
Supplement: Supplementary file 1 [file toxics-11-00952-s001.zip › toxics-2692577-supplementary.pdf]

Supplementary Materials: Table

Table S1. Primer sequences employed.

mRNA	Sequences
H2ac19 F	ACAAGCTGCTGGGCAAAGTGAC
H2ac19 R	CTTATGGTGGCTCTCCGTCTTCTTG
Spp1 F	AAACACACAGACTTGAGCATTC
Spp1 R	TTAGGGTCTAGGACTAGCTTGT
Slc34a1 F	CCTGAGGAATCACAGTCTCATT
Slc34a1 R	CCACGAAGATATGGTTGCATTT
Nphs1 F	CATCCAGGAGTGTTTTTCTTCG
Nphs1 R	CAGGAACTCTGGAGGGTATAAC
Lpl F	CCTGATGACGCTGATTTTGTAG
Lpl R	CAATGAAGAGATGAATGGAGCG
Kap F	TCTCCTGTTCACTGGTTGCCTTAAC
Kap R	GGAAAGCCACAGTCAGACCACAG
Umod F	ATATTTACAACTTGACAGCGCC
Umod R	TGATGTTGGAGTCCTGTTTACA
Keg1 F	AGAAGAGCCAGAAGTTCATTGA
Keg1 R	TCTGAACAGTGAAGTTAGCCTT
Cyp2e1 F	CCAACTCTGGACTCCCTTTTAT
Cyp2e1 R	ACGCCTTGAAATAGTCACTGTA
GAPDH F	ACAACTTTGGTATCGTGGAAGG
GAPDH R	GCCATCACGCCACAGTTTC

miRNA	Sequences
miR-19b-1-5p F	GTTAGTTTTGCAGGTTTGCATCC
miR-27b-3p F	AGGTGCAGAGCTTAGCTGAT
miR-11987 F	CGAGGAATCTCTGGTGGAGG
miR-128-1-5p F	GTTGGATTCGGGGCCGTAG
miR-466b-5p F	TCATTAGTGCTGAGTATGTGTGTGT
U6 F	CTCGCTTCGGCAGCACATATACT
U6 R	ACGCTTCACGAATTTGCGTGTC

circRNA	Sequences
mmu_circ_0006286F	TGCTGCGTCCAAACATAAACAACTG
mmu_circ_0006286R	GGGCTGCTGGCACTCACATTG
mmu_circ_0003133F	GAGCACGCTATGAGAGATACAGTGG
mmu_circ_0003133R	ACCAAAACTGCAAGGAGGAAGGAC
mmu_circ_0013551F	CAACAAAGAGGCTGAAGGGAGAGAG
mmu_circ_0013551R	GGAGATTGGCAGACGGATGACAG
mmu_circ_0013415F	CAGTCGCCTCGGTGCCTATAATG
mmu_circ_0013415R	GCCATCCTGATCCAGAACTTCATCC
mmu_circ_0003030F	CAGTTCCCAACCTTTGAGCAATGTC
mmu_circ_0003030R	GCGTAGACCTCCATGCACCAAG
mmu_circ_0000284F	ATGACACCTGAGCAGGAGGAGAC
mmu_circ_0000284R	CTCACTCACTAAGCGGTAACCACTG
mmu_circ_0000231R	GCAGTCTCTCATCAGGGCATATCAG
mmu_circ_0000231R	ACGGAGGAGTCCAGATCAGCATC

lncRNA	Sequences
lnc Gm31786 F	AAGCGGCATGATGGTTGGAAGAG
lnc Gm31786 R	GGGTGTGGCAGTAAAGGTGAACTC
lnc 4921504A21Rik F	ACACAGCAAGAGAGGCAGTTTCAG
lnc 4921504A21Rik R	GCAGCAGAAGGCAGGGTTCAC
lnc Rsf1os2 F	CACGCATCTGGAGATCCCAAGTC
lnc Rsf1os2 R	GGCTGTGTGAACCCAAGGAGAAC
lnc Cd55os F	CAGCAGCAGTGGTGGTGATGAC
lnc Cd55os R	CCAAATCCTGGCATGGTCTCTTCC
lnc Gm31786 F	AAGCGGCATGATGGTTGGAAGAG
lnc Gm31786 R	GGGTGTGGCAGTAAAGGTGAACTC
lnc 1700009J07Rik F	AGCCACCACTAAGCAAGCACATC
lnc 1700009J07Rik R	AGCCTCCACATGACAGGACCAG
lnc Tmem134 F	AGCCTCCACATGACAGGACCAG
lnc Tmem134 R	ACAAAGAAGATGGCACTGGAGACAC
lnc Neat1 F	TCCTCTTCTGTGGTCTCACTCTTCC
lnc Neat1 R	ACTCCAGCACCGACTCTACATCC
lnc Meg3 F	AGGGAAGCAGAGGTCGCCAAG
lnc Meg3 R	AGCACAGTGGAGCCAGGAGTC
lnc Smim41 F	CTGTCTTGCCTGATGCGGTATCTG
lnc Smim41 R	ATGCTGGAACTGTTGCTGGATGG
lnc Gm6729 F	TGCCTGCCACCTGAGGAAGAC
lnc Gm6729 R	CTACGCTCTGTGTTTGTCTCTGGAG

Figures and Figure legends

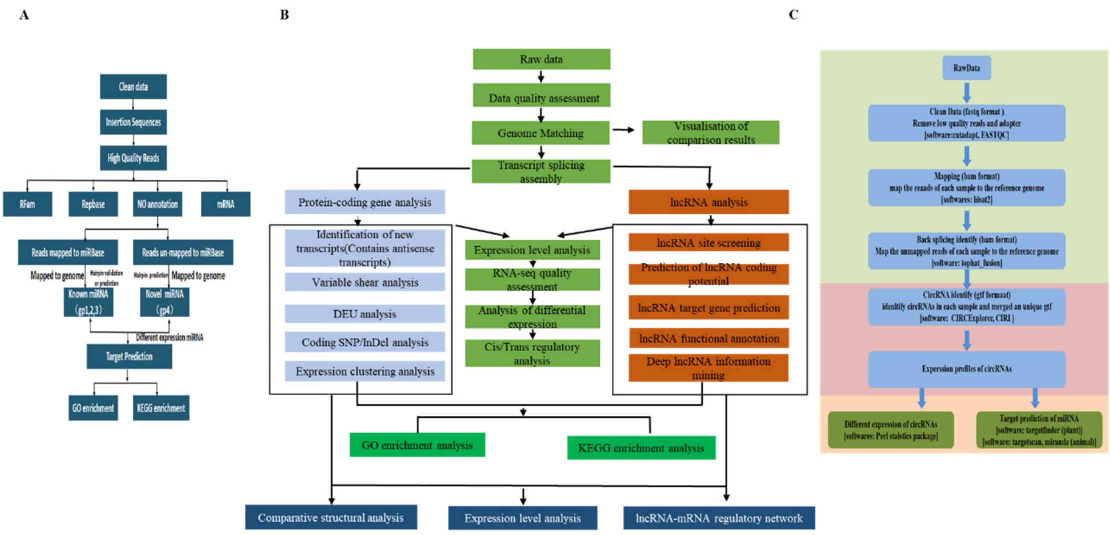

Figure S1. Bioinformatic methods for the analysis of mRNAs, miRNAs, circRNAs and lncRNAs

(A) Flow chart of differentially expressed miRNA analysis. (B) Flow chart of differentially expressed lncRNA and mRNA analysis. (C) Flow chart of differentially expressed circRNA analysis.
